# How the *BRAF* V600E Mutation Defines a Distinct Subgroup of Colorectal Cancer: Molecular and Clinical Implications

**DOI:** 10.1155/2018/9250757

**Published:** 2018-11-26

**Authors:** Catherine E. Bond, Vicki L. J. Whitehall

**Affiliations:** ^1^Conjoint Gastroenterology Laboratory, QIMR Berghofer Medical Research Institute, Brisbane, Queensland, Australia; ^2^School of Medicine, The University of Queensland, Australia; ^3^Pathology Queensland, Brisbane, Queensland, Australia

## Abstract

The *BRAF* oncogene is an integral component of the MAP kinase pathway, and an activating V600E mutation occurs in 15% of sporadic colorectal cancer. This is an early event in serrated pathway tumourigenesis, and the *BRAF* V600E has been commonly associated with the CpG island methylator phenotype, microsatellite instability (MSI), and a consistent clinical presentation including a proximal location and predilection for elderly females. A proportion of the *BRAF* mutant lesions remain as microsatellite stable (MSS), and in contrast to the MSI cancers, they have an aggressive phenotype and correlate with poor patient outcomes. Recent studies have found that they have clinical and molecular features of both the *BRAF* mutant/MSI and the conventional *BRAF* wild-type cancers and comprise a distinct colorectal cancer subgroup. This review highlights the importance of the *BRAF* mutation occurring in colorectal cancer stratified for molecular background and discusses its prognostic and clinical significance.

## 1. Introduction

In colorectal cancer, the presence of a *BRAF* mutation can be associated with an aggressive phenotype and is a key prognostic biomarker for poor outcome particularly in late-stage disease. *BRAF* is a protein kinase and part of the MAP kinase signalling cascade which involves transduction of a growth signal from the cell membrane to the nucleus via a chain of protein kinases and is responsible for cellular proliferation and survival. An activating hotspot mutation occurs at V600E and results in constitutive MAPK signalling and uncontrolled cellular growth. The *BRAF* V600E mutation occurs early in tumourigenesis and is highly correlated with the serrated neoplasia pathway of colorectal cancer. This pathway describes progression of a serrated precursor lesion, often followed by the onset of epigenetic instability involving promoter methylation and silencing of key tumour suppressor genes, and accounts for 15%-20% of sporadic colorectal cancer [1, 2].

A proportion of *BRAF* mutant lesions will methylate a DNA mismatch repair gene, *MLH1*, which leads to the onset of microsatellite instability (MSI) [3]. The reported incidence of BRAF mutant lesions that develop MSI ranges from 46% to 75% [4–8], and these BRAF mutant/MSI cancers have been well characterized to show typical clinicopathological features such as a predilection for elderly females and a proximal location. The remaining *BRAF* mutant cancers that do not methylate *MLH1* to develop MSI stay as microsatellite stable (MSS). This latter *BRAF* mutant/MSS cancer subgroup has not been as well studied, but is known to particularly associate with a poor patient outcome.

*BRAF* mutant/MSS cancers form a distinct colorectal cancer entity that shares clinical and molecular features with both *BRAF* mutant/MSI serrated pathway cancers and the *BRAF* wild-type cancers of the conventional pathway [9–11]. This latter pathway involves the previously well-defined series of genetic aberrations such as *APC* mutation and chromosomal instability and accounts for the majority of colorectal cancer [12].

Recent studies have also found that presence of the *BRAF* mutation has direct implications for clinical management as along with TNM stage, *BRAF* mutational status was the only molecular variable that independently accounted for poor survival [13], and studies have found that *BRAF* mutant cancers are refractory to anti-EGRF therapy [14, 15]. Although the relevance of mutant *BRAF* in the clinical setting is increasingly being acknowledged, the relatively low frequency of its occurrence requires further studies and larger experimental cohorts to secure its mutational status as a definitive biomarker for colorectal cancer.

This review will explore our current understanding of *BRAF* mutant cancers with respect to presence or absence of MSI.

## 2. *BRAF* Gene and the MAP Kinase Pathway

**Figure 1:**
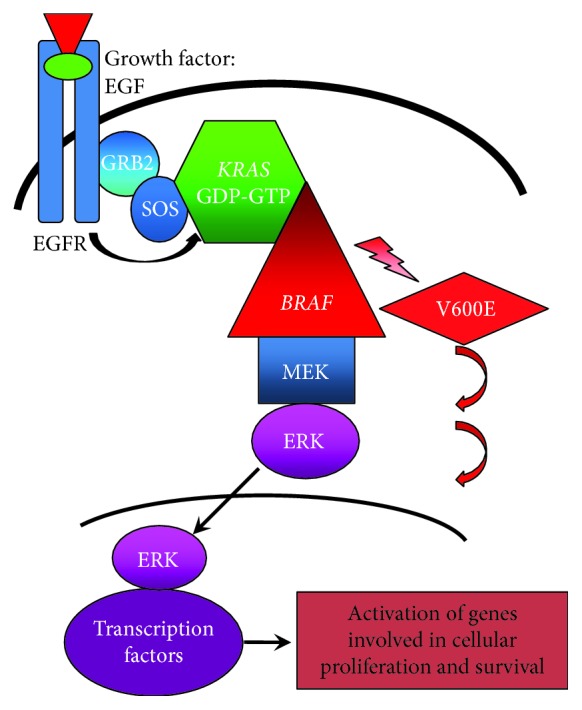
Diagram of the mitogen-activated protein kinase pathway. A signalling epidermal growth factor binds to the receptor (EGFR) on the cell surface causing its phosphorylation and activation. The activated signal is passed to scaffolding proteins (GRB2 and SOS) which in turn promotes the removal of GDP from membrane-bound KRAS. KRAS then binds GTP, allowing its activation, and undergoes conformational change to bind and phosphorylate BRAF. The signalling cascade continues through MEK and ERK. Activated ERK translocates to the nucleus where it recruits transcription factors involved in cellular survival and growth. The V600E BRAF mutation allows for constitutive activation of BRAF and continuation of downstream signalling regardless of upstream regulation.

The *BRAF* (v-raf murine sarcoma viral oncogene homolog B) gene encodes a protein that belongs to the Raf family of serine/threonine protein kinases. It is an integral component of the MAP (mitogen-activated protein) kinase cascade. This signal transduction pathway is initiated by epidermal growth factor ligands binding to and activating receptor tyrosine kinases (RTK) at the cell membrane. Adaptor proteins, GRB2 and SOS, are then sequentially recruited to stimulate the release of GDP from KRAS which permits binding of GTP to activate KRAS. KRAS which is located at the plasma membrane then undergoes conformational change to enable its direct binding and recruitment of cytosolic BRAF [16] which upon stimulation forms an active dimer. This in turn phosphorylates and activates MEK and then ERK. Activated ERK subsequently translocates to the nucleus where it can stimulate transcription factors involved in the promotion of cellular proliferation and survival (Figure 1). The MAPK pathway is not a linear signal transduction cascade; additional scaffolding proteins, such as KSR1 assemble and maintain the cellular location of the cascade components to aid efficiency of the signalling pathway. In addition, a negative feedback mechanism exists whereby phosphorylated ERK may inhibit upstream components and pathway negative regulators, SPRY and DUSP, are expressed upon signal activation to facilitate controlled output.

In addition to BRAF, two other Raf kinase family isoforms exist: ARAF and CRAF (or *RAF-1*). Each varies in cell-specific expression and biochemical ability to be activated by oncogenic KRAS. BRAF confers the greatest potential of stimulation due to its constitutively phosphorylated S445 within the regulatory (N) region which becomes activated upon recruitment to the plasma membrane via KRAS; CRAF and ARAF require the kinase Src for full activation [17–19]. Additionally, BRAF has the highest affinity and efficiency for MEK binding [20] which confirms it as the strongest RAF isoform in driving this signalling cascade.

### 2.1. BRAF Mutations

Across several cancer types, the vast majority of *BRAF* mutations occur at the V600 amino acid residue where a transversion of a thymidine to adenosine occurs at nucleotide 1799, resulting in conversion of valine to glutamate [2]. This mutation results in a negative charge adjacent to the T599 phosphorylation site which is sufficient to cause constitutive BRAF activation independently of upstream signalling [2]. The V600E mutation confers approximately a 500-fold greater activity than wild-type BRAF and can induce transformation in cell line models [2]. Across all cancer types, the *BRAF* V600E mutation occurs in 8% of cancers, with melanoma having the highest rate of *BRAF* V600E mutations at 66%, followed by papillary thyroid cancer (53%) and serous ovarian cancer (30%) [2, 17]. In colorectal cancer, the *BRAF* V600E mutation accounts for the vast majority of all *BRAF* mutations and occurs in approximately 15%-20% of sporadic cases [1, 2].

Typically, BRAF mutations have been identified using targeted techniques for the V600 mutation hotspot such as an allelic discrimination assay that employs allele-specific primers to identify either wild-type or mutant alleles [21]. Advancing technology has more recently allowed for efficient genome-wide analyses such as the MSK-IMPACT platform which uses capture-based next-generation sequencing technologies to cover exonic regions of multiple genes [22]. Utilising this form of technology has highlighted the presence of non-V600 *BRAF* mutations that occur in cancers including colorectal cancer.

A study of almost 10,000 metastatic CRC that were sequenced with MSK-IMPACT showed that non-V600 *BRAF* mutations occurred in 2.2% of cases, the vast majority being found in MSS cancers, and accounted for 22% of all BRAF mutations [23]. Interestingly, these non-V600 mutant cancers had a very different clinical presentation to V600 mutant cancers as they had a predilection for younger male patients, occurred more commonly on the left side, and were less likely to occur in high-grade cancers. Additionally, non-V600 mutant cancers were associated with more favourable overall survival rates compared to both *BRAF* V600 mutant and wild-type cancers [23].

*BRAF*-activating mutations have been classified based on their dependence on KRAS for signalling and whether they act as a monomer or dimer. Class 1 substitutions include the V600 mutations and are constitutively active as monomers or active dimers, but the latter is dependent on KRAS; class 2 mutants are constitutively active as dimers independent of KRAS and consist of kinase active non-V600 mutation types [24]. Recently, a third class of BRAF mutants were identified that have impaired or no kinase activity, can function as a dimer, and are dependent on activated KRAS. MEK and ERK are still phosphorylated by these class 3 mutations but to a lesser degree than class 1 and 2 kinase-activating mutations [25]. In recent analyses of non-V600 mutations, class 3 or kinase-impaired mutants were over twice as likely to occur than class 2 mutants [23, 26].

The class of BRAF mutation has clinical implications regarding the type of BRAF inhibitor prescribed as it has been found that FDA-approved BRAF inhibitors vemurafenib and dabrafenib are only effective against class 1 monomer-type mutations (V600 mutations) and not the class 2 mutations that function as dimers due to an elevated drug affinity for a site within the first dimer which causes an allosteric effect and subsequent reduction of binding to the second dimer [24]. However, resistance to these BRAF inhibitors in class 1 mutants can occur; for example, upregulated protein expression of BRAF from gene amplification events can cause increased BRAF homodimerization [24]. Next-generation BRAF inhibitor compounds, such as LY3009120 and BGB659, are aimed at overcoming the problems associated with dimerization and in preclinical models have been shown to bind to class 1 monomers and both dimers equally in class 2 mutants, as well as all RAF isoforms [24, 27, 28]. Class 3 BRAF mutants were found to concurrently express high levels of phosphorylated EGFR which rendered them sensitive to EGFR antibody treatment which is in contrast to class 1 and 2 mutants and may provide an effective avenue of therapy for patients with this mutation type [25, 26].

The class 1 BRAF V600E mutation and also class 2 mutations allow for constitutive activation of the pathway; therefore, a concurrent KRAS mutation is redundant which is indicated by the mutual exclusivity of these two mutation types occurring in a single cancer [1, 29]. Interestingly, it was found that the V600E mutation conferred more elevated levels of phosphorylated MEK compared to mutant KRAS, which suggests the potency of this mutation in upregulation of the MAPK pathway [24]. In contrast, class 3 BRAF mutations are dependent on activated KRAS and can coexist and even synergize with a KRAS mutation [25]. Although occurring exclusively, mutation of either RAS or BRAF is commonly found in colorectal, thyroid, and ovarian cancers and melanoma [1, 2, 30, 31] which suggests that deregulation of the MAPK pathway at either level confers a selective advantage for these cancer types [2].

Although relatively rare, the presence of non-V600E mutations in colorectal cancer has important implications for clinical management with the choice of therapy and indicates that more gene-wide mutation screening regimes are warranted. The remainder of this review will focus primarily on the more abundant *BRAF* V600E mutation and that which confers a significant clinical impact on colorectal cancer.

## 3. The *BRAF* V600E Mutation in Colorectal Cancer

Colorectal cancer is a heterogeneous disease and is comprised of multiple molecular pathways. The *BRAF* mutation is most closely associated with the serrated neoplastic pathway in which a serrated type polyp, either a sessile serrated adenoma (SSA) or a traditional serrated adenoma (TSA), acquires defined molecular aberrations leading to cancer [32].

### 3.1. Frequency of the BRAF V600E Mutation in Colorectal Cancer Precursor Lesions

The *BRAF* V600E mutation in colorectal cancer is an early event as indicated by its presence in one of the earliest forms of premalignant lesion, the hyperplastic or serrated aberrant crypt foci (ACF) (63%), but is rarely present in nonserrated ACF (6%) [33]. It is understood that the microvesicular hyperplastic polyps (MVHPs) are the precursor to sessile serrated adenomas (SSAs) which are the most prevalent type of serrated adenoma due to the high prevalence of the *BRAF* mutation in both (70-95% and 78-85%, respectively) [32, 34–36]. The *BRAF* mutation is present in traditional serrated adenomas (TSAs) which are the least frequently occurring type of colorectal polyp (33-66%), but rarely in conventional adenomas (0.4-5%). Interestingly, *KRAS* mutations have been predominantly found in nonserrated ACF (42%) and goblet cell hyperplastic polyps (43-54%) [33, 34, 37–40]. These findings demonstrate that *BRAF* mutation is an exclusive feature of the serrated pathway due to its frequency in the majority of serrated polyps but rare presence in conventional adenomas [34]. Similar to its presence in serrated-type polyps, *BRAF* mutations have been found in benign melanocytic nevi [41, 42] which suggests that a *BRAF* mutation in the initiation of cancer is necessary but insufficient to cause progression to a malignant state and that other molecular aberrations are required for this critical step. This is supported by the finding that *BRAF* V600E mutant organoids when orthotopically injected into a murine colon were unable to induce tumourigenesis until further serrated neoplasia-type genetic perturbations were introduced [43].

### 3.2. BRAF V600E Mutation and Methylation

DNA methylation at CpG islands within promoter regions of tumour suppressor genes has been associated with transcriptional silencing and provides a selective advantage for cancer proliferation [44, 45]. PCR-based methylation screening techniques were initially developed in order to determine the methylation status of multiple CpG sites across large sample cohorts [46, 47]. It was found that although incremental increases in methylation did occur in certain genes as a function of age in normal mucosa, there were cancer-specific methylation events occurring within a distinct subset of genes and the CpG island methylator phenotype (CIMP) was termed [46]. Methylation of the DNA mismatch repair gene, *MLH1*, had previously been determined to lead to MSI [3], and CIMP correlated with the presence of MSI as well as proximal cancer location [46]. However, it was observed that methylation events accumulated more across a progressive continuum that may have been more related to age-dependent changes [48]. In addition, corresponding clinical and molecular parameters were found to be more attributable to the mutator or MSI phenotype rather than an epigenetic phenotype [48, 49]. A subsequent panel of methylated markers devised from interrogating almost 200 CpG sites across 300 colorectal cancers and using a sensitively quantitative MethyLight technology was able to detect a bimodal distribution of the methylator phenotype which was specific for *BRAF*-mutated cancers reliably segregating with CIMP-high [50]. This panel has been widely utilised and provides a readily available platform to indicate the methylation status of large numbers of samples. Recent studies that have utilised genome-wide methylation arrays have confirmed that those cancers with the most frequent and widespread methylation do significantly correspond with the BRAF mutation and MLH1 methylation [51, 52]. Methylation was observed to occur as a continuum at several sites; however, 4 and 5 distinct methylation-based clusters were identified [51, 52], and cancers with the most methylated genomes not only were BRAF mutant and MSI but also classified as CIMP-high using the more recent CIMP-denoting markers [50] which supports the utility of this panel.

As discussed above, serrated pathway cancers mutate BRAF early in tumourigenesis due to their presence in the earliest form of precursor lesions. Methylation events also occur early and have been found in 42-51% SSAs [35, 53] and 45-64% of TSAs [54, 55].

Mechanistically, it has been shown in *BRAF* V600E mutant colorectal cancer and melanoma cell lines that overactivation of the MAPK pathway phosphorylates and upregulates MAFG which functions as a transcriptional repressor. MAFG can bind to the promoters of methylation-prone genes via DNA consensus sites then recruit components of a repressor complex including a DNA methyltransferase which results in hypermethylation and repression of these genes [56, 57]. Further, we have found in vivo that presence of the *Braf* V600E mutation in an intestine-specific murine model of serrated colorectal neoplasia was sufficient to cause considerable DNA methylation from 5 months of age in a gene-specific manner. This indicates that persistent *BRAF* mutant signalling is causal in the induction of wide-ranging DNA methylation events in a temporal pattern. In addition, there was extensive intestinal hyperplasia from as early as 10 days post-*Braf* mutation induction [58].

### 3.3. The BRAF V600E Mutation in the Serrated Pathway

**Figure 2:**
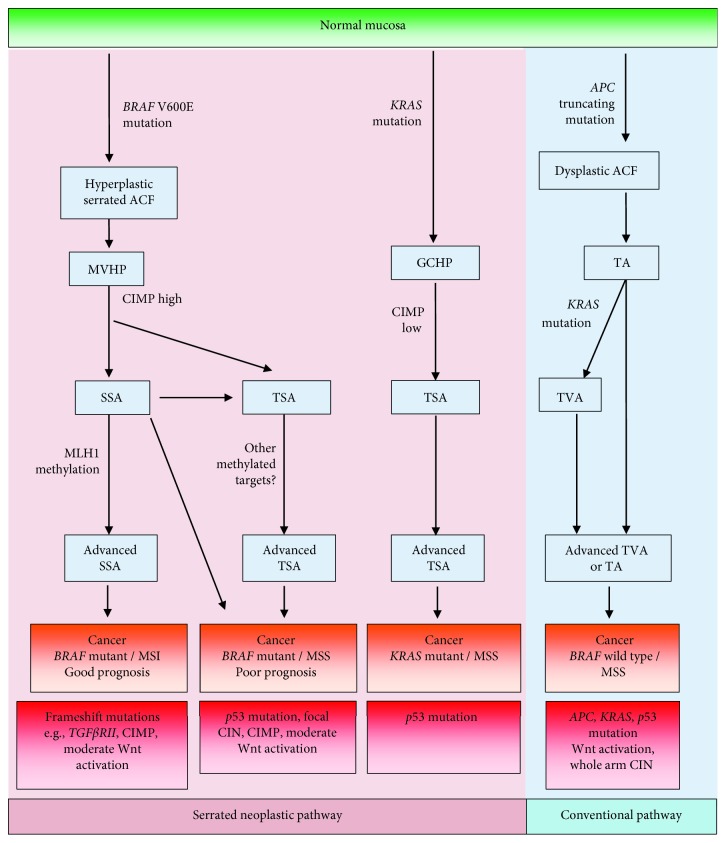
Diagram of the serrated and conventional pathways of colorectal cancer. Bold text indicates the pathways leading to *BRAF* mutant cancers. ACF: aberrant crypt foci; GCHP: goblet cell hyperplastic polyp; MVHP: microvesicular hyperplastic polyp; SSA: sessile serrated adenoma; TSA: traditional serrated adenoma; TA: tubular adenoma; TVA: tubulovillous adenoma.

From findings associating the key genetic events of *BRAF* mutation and hypermethylation events being present in the earliest form of serrated precursor lesions and not in conventional-type adenomas, a continuum of serrated tumourigenesis from early serrated ACF to MVHP then to SSA was proposed [35, 37, 59]. SSAs may then undergo methylation and loss of key tumour suppressors such as *p16* and *MLH1* which coincide in regions of dysplasia to drive transformation to carcinoma [59]. Epigenetic loss of the DNA repair gene, *MLH1*, results in widespread frameshift mutations in DNA repeat tracts termed microsatellite instability (MSI) (Figure 2).

## 4. *BRAF* Mutant/MSI Cancers

*BRAF* mutant/MSI cancers are the most well-characterized subgroup of the serrated pathway due to the consistent findings of several clinicopathological and molecular features. Previous studies have found them to arise in the proximal colon, to more commonly affect females at an older age and associate with a favourable patient outcome [60–64]. Histologically, they show poor differentiation, are mucin-producing, and contain tumour-infiltrating lymphocytes [64, 65]. The latter feature may be indicative of an immune response to the high rate of truncated proteins formed as a result of multiple frameshift mutations induced by MSI [66] and may underlie their favourable outcome. The stimulation of an immune response in patients with MSI-type cancers is evident from increased infiltration of CD8 lymphocytes and greater expression of PD-L1 checkpoint protein at the invasive front than in MSS-type cancers. Furthermore, patients with MSI cancers have shown significantly greater progression-free survival when treated with immunotherapy checkpoint inhibitors [67].

Molecular features include a high frequency of the methylator phenotype (CIMP) (80%) [9], infrequent loss or mutation of *APC* and *p53* genes, and a minimal extent of chromosomal aberrations [9, 11, 63–65, 68–70]. Methylation of the DNA repair gene, *MGMT*, which has been associated with G > A transition mutations in *p53*, was most frequent in *BRAF* mutant/MSI cancers compared to *BRAF* mutant/MSS cancers. Although *BRAF* mutant/MSI cancers did have the greater frequency of *p53* mutant G to A transitions, these were not associated with concurrent *MGMT* methylation in this series [9]. *PIK3CA* is a component of the PI3K signalling pathway and is frequently mutated at hotspots in either exon 9 or 20 in colorectal cancer [71–73]. Overall, *PIK3CA* mutations have been associated with *KRAS* mutant, *MGMT* methylated, and CIMP cancers [73]; however, *PIK3CA* mutations in the catalytic exon 20 hotspot were found predominantly in *BRAF* mutant/MSI cancers [73].

The Wnt signalling pathway is commonly activated in conventional colorectal cancer via truncating APC mutations [8]. The *BRAF* mutant serrated pathway rarely mutates APC in this manner; however, activation of the Wnt pathway can occur through alternative mechanisms as nuclear beta-catenin is evident in approximately half of the serrated dysplastic polyps and cancers studied [74]. More moderate missense APC mutations occur in *BRAF* mutant/MSI cancers which may influence the Wnt signal, and it was found that 87% of *BRAF* mutant/MSI cancers mutate RNF43 which is a negative regulator of the Wnt signal [74, 75]. In addition, alternative aberrations found in *BRAF* mutant/MSI lesions to activate the Wnt pathway include methylation of Wnt antagonists such as AXIN2 [76]; and SFRP4, DKK1, and WNT5A, the latter of which regulates the noncanonical Wnt pathway, were found to be methylated in *BRAF* mutant/MSI/CIMP-positive cancers [77, 78].

## 5. *BRAF* Mutant/MSS Cancers

SSAs may methylate an epigenetic target other than *MLH1* and result in *BRAF* mutant/MSS cancers [59]. A recent study of 200 TSAs found that all lesions retained *MLH1* expression which associates with an MSS phenotype and therefore indicates TSAs may progress to *BRAF* mutant/MSS cancers [54]. In addition, this study found residual presence of an SSA in approximately 30% of TSAs which suggests SSAs that do not methylate *MLH1* could progress via a TSA to *BRAF* mutant/MSS cancer (Figure 2).

The *BRAF* mutant/MSS cancers have not been as thoroughly studied as the *BRAF* mutant/MSI cancers. However, they have been found to consistently associate with a detrimental patient outcome [4–6, 79, 80], and recent investigations have identified them as a unique subgroup of colorectal cancer [9–11, 81]. Although they derive from a serrated type polyp due to presence of the *BRAF* mutation, they diverge from the more well-known *BRAF* mutant/MSI serrated pathway cancers with the development of distinguishing clinical features and genetic aberrations that represent features from both the serrated and conventional pathways.

Clinically, the *BRAF* mutant/MSS cancers are similar to the *BRAF* mutant/MSI cancers as they frequently occur in the proximal colon but also resemble *BRAF* wild-type cancers in terms of equal gender distribution and a younger age of onset and they more commonly present at advanced stages [9–11]. Another study found that of the three subgroups, *BRAF* mutant/MSS cancers were the most likely to present at stage IV, of high grade, and more distally located than *BRAF* mutant/MSI cancers [81]. Overall, the above investigations indicate that the *BRAF* mutant/MSS cancers clinically represent a distinct cancer subtype.

Histologically, *BRAF* mutant/MSS cancers are similar to *BRAF* mutant/MSI cancers in terms of being mucinous and poorly differentiated [65, 82]. However, they do show more adverse morphological features which corresponds with their aggressive phenotype such as frequent tumour budding, a lack of tumour-infiltrating lymphocytes, frequent lymphatic, perineural, and venous invasion and increased lymph node metastases compared to *BRAF* mutant/MSI and *BRAF* wild-type cancers [81–83].

In colorectal cancer, immunohistochemical markers *CDX2*, *CK20*, and *CK7* are frequently used to ascertain whether a metastasis is colorectal in origin. As *BRAF* mutant/MSS cancers frequently metastasize, clarification of the expression of these markers is important for accurate diagnosis of the primary tumour site. Reduced levels of *CDX2* staining which is correlated with MSI, *BRAF* mutation, and CIMP [84, 85] were also found in the *BRAF* mutant/MSS cancers, which suggests that epigenetic silencing associated with CIMP and the *BRAF* mutation may be contributing to downregulation of *CDX2* [86]. *CK20* was retained in *BRAF* mutant/MSS [81] as typically seen in *BRAF* wild type, but lost in *BRAF* mutant/MSI cancers [81, 84, 85, 87]. *CK7* is minimally present in colorectal cancer [88, 89] but was significantly more frequently upregulated in *BRAF* mutant/MSS cancers compared to the other cancer subtypes [81]. Interestingly, *CK7* has been found in regions of tumour budding [90] indicating progression of disease [91], which is also more prevalent in the aggressive *BRAF* mutant/MSS cancers [82].

### 5.1. Molecular Features of BRAF Mutant/MSS Cancers

*BRAF* mutant/MSS cancers have multiple genetic aberrations that are representative of typical changes associated with both serrated and conventional pathways. *BRAF* mutant/MSS cancers often display hypermethylation events (at 60%) compared to the infrequent occurrence in conventional pathway cancers (3%) [9, 10]. Therefore, this demonstrates that CIMP is prevalent in all *BRAF* mutant cancers of the serrated pathway, but at a higher frequency in MSI (70–80%) than MSS (60%) cancers [9, 11].

*P53* mutation, which associates with advanced stage, has been correlated with conventional pathway cancers as it was uncommon in MSI cancers [92–95]. *BRAF* mutant/MSS cancers of the serrated pathway have been found to have a comparably high rate of *p53* mutation as the *BRAF* wild-type cancers, whereas *BRAF* mutant/MSI cancers were confirmed to have a low rate of mutation [9]. This finding of a similar extent of mutation of this important tumour suppressor gene in *BRAF* mutant/MSS and *BRAF* wild-type cancers was the first report of molecular similarity occurring between the serrated and conventional pathways and provided evidence of a molecular overlap between the two. In a subsequent murine model of *BRAF* mutation-induced tumourigenesis, it was found that the addition of mutant *p53* to mutant *BRAF* resulted in formation of the same number of TSAs compared to the *BRAF* mutation alone, but invasive cancers were more frequent in the intestines of mice harbouring mutant *p53* and *BRAF* [96]. Additionally in this murine model, mutant *p53* was infrequently present in *BRAF* mutant TSAs with low-grade dysplasia but substantially increased in those with high-grade dysplasia [96]. This study also demonstrates the involvement of mutant *p53* in *BRAF* mutant serrated pathway tumourigenesis and further supports its role in the progression of *BRAF* mutant serrated lesions to cancer rather than in their initiation [96].

Similarly to *BRAF* mutant/MSI cancers, *BRAF* mutant/MSS cancers do not typically truncate APC but still show moderately frequent activation of the Wnt pathway [74]. This may occur due to missense mutations of the Wnt pathway regulator, RNF43, which occurs at 24% in these cancers [75]. Additionally, methylation of several Wnt antagonists such as Dkk2 and Sfrp1 were evident in a murine model of this cancer type [58].

*BRAF* mutant/MSS cancers have been found to have a comparably high rate of chromosomal instability (CIN) as *BRAF* wild-type cancers [10, 11]. This has been demonstrated by loss of heterozygosity analysis at key loci where deletion events associated with late- compared to early-stage *BRAF* mutant/MSS cancers overall and at loci on 5q, 17p, and 18q, and loss at 18q and 8p correlated with worse survival [10]. This indicates that CIN contributes to progression of disease and may relate to the poor outcomes associated with *BRAF* mutant/MSS cancers [10]. Genome-wide single-nucleotide polymorphism (SNP) arrays also found a frequent and similar rate of copy number aberrations occurring in *BRAF* mutant/MSS and *BRAF* wild-type cancers [11]. However, a notable difference between the two cancer subgroups was that a “focal pattern” indicated by a high proportion of small, targeted copy number aberrations (CNAs) was a feature of the *BRAF* mutant/MSS cancers and a “whole chromosome arm” pattern featuring predominantly chromosome arm-length CNAs was evident in *BRAF* wild-type cancers. The different patterns of CIN between the two MSS cohorts stratified by *BRAF* mutation status were a novel finding. This may have implications relating to differences in the causes of CIN as well as potentially impacting clinical outcomes relevant to the molecular subtype of CRC [11].

CIMP-positive, *BRAF* mutant/MSS cancers were found to harbour a hotspot mutation at R132 in *IDH1* [97] which causes CIMP in glioma [98]. This indicates that in a proportion (4/45, 9%) of this particular *BRAF* mutant colorectal cancer subgroup, *IDH1* mutation may contribute to CIMP. Furthermore, all *IDH1* mutant cancers were found to have a more closely related methylation profile compared to other cancers which may reflect a similar mechanism of epigenetic instability [97].

From the above findings of the molecular characterization of *BRAF* mutant/MSS cancers compared to other colorectal cancer subgroups, the compounding effects of the presence of CIMP, a focal pattern of CIN, mutant *p53*, and methylated genetic targets may be contributing to their advanced clinical stage and their association with unfavourable patient outcomes.

## 6. *BRAF* Mutation and Gene Expression Subtypes

Recently studies have analysed gene expression data to stratify colorectal cancers into distinct subtypes. From a comprehensive framework using aggregated gene expression data from multiple datasets, Guinney et al. studied almost 2000 colorectal cancers with known *BRAF* mutational status that included 200 *BRAF* mutant cancers and identified four consensus molecular subtypes (CMSs). The majority of the *BRAF* mutant cancers (at 70%) grouped into CMS1 which was enriched with cancers positive for MSI, methylation, activated immune pathways, and a high propensity for females. The next highest proportion of *BRAF* mutant cancers (at 17%) were grouped with CMS4 which consists of MSS cancers with upregulation of genes involved in epithelial-to-mesenchymal transition (EMT) and worse survival rates [99]. Overall, these CMS1 and CMS4 groupings are fairly consistent with the above described molecular characteristics and phenotypes of *BRAF* mutant cancers stratified for MSI status.

A recent study that analysed transcript expression in approximately 200 *BRAF* V600E mutant cancers, many of which were from the same publically available datasets as the study by Guinney et al. [99], found that *BRAF* mutant cancers grouped into two distinct molecular entities (BM1 and BM2) [100]. This dedicated analysis on BRAF mutant cancers only is aimed at improving characterization of the heterogeneity found within BRAF mutant cancers. Although the two groupings did not significantly associate with any clinical characteristics, trends were found for an improved prognosis and MSI associating with BM2, and poorer survival and an EMT signature were features of BM1. Further, BM1 cancers had a *KRAS*-related expression signature whereas BM2 cancers correlated with cell cycle and checkpoint-associated gene expression. Due to these findings, the authors suggested that the two subtypes would benefit from different targeted therapies and, with the inclusion of greater cohort numbers from further studies, the potential to translate to clinical application could be met [100].

## 7. Prognostic Implications for *BRAF* Mutant Cancers

There is widespread heterogeneity of genetic, clinical, and morphological features occurring in colorectal cancer, even when segregated into their respective pathways of origin. The remainder of this review discusses the *BRAF* mutation in terms of prognostic and clinical implications, and although this mutation occurs in varied genetic backgrounds, its presence can influence clinical practice and choice of therapy.

The *BRAF* V600E mutation is increasingly being recognized as a negative prognostic factor particularly in late-stage colorectal cancer independent of associated clinicopathological variables [101] and is one of the most consistent molecular markers that confer a poor outcome. This effect is particularly apparent with presence of the *BRAF* mutation in a non-MSI background [4–6, 14, 79, 80, 102–104], and the significantly poorer survival rates of *BRAF* mutant/MSS cancers compared to other colorectal cancer subtypes are often maintained after multivariable-adjusted analyses [5, 6, 102, 104].

Early-stage *BRAF* mutant/MSI cancers as discussed above have an activated immune profile with lymphocytic infiltration which could be causal to their good prognosis [62]. The frequency of late-stage MSI cancers is lower than MSS cancers which reflects the lower metastatic risk of MSI cancers [105], however the prognostic benefit conferred by MSI was lost in the metastatic setting where *BRAF* mutant/MSI cancers had similarly poor survival rates as *BRAF* mutant/MSS cancers [106, 107]. This indicates that the detrimental prognosis seen in late-stage MSI cancers is driven by presence of the BRAF mutation [106–108].

Several studies have reported that *BRAF* mutant cancers are more likely to metastasize to the peritoneum rather than the lung or liver [13, 107, 109, 110]. Poorer survival has been associated with peritoneal spread which may reflect more the propensity of aggressive *BRAF* mutant cancers to metastasize to this region [110]. Furthermore, patients with late-stage *BRAF* mutant cancers were less likely to have a metastasectomy most likely due to the higher incidence of peritoneal involvement, and survival following metastasectomy was significantly shorter for patients with BRAF mutant compared to *BRAF* wild-type cancers [109].

Two studies that examined large cohorts of stage II and III CRC [4, 79] reported that MSS cancers with a *BRAF* mutation had a worse prognosis compared to those with a *KRAS* mutation [4], and this was independent of disease stage and adjuvant treatment [79]. A further study examining overall survival associated with post-lung metastasectomy found that it was significantly worse for *BRAF* mutant compared to *KRAS* mutant and wild-type cancers [111].

However, a recent study that stratified a large cohort of stage III cancers for MMR status and *BRAF* and *KRAS* mutations found that MSS cancers with a *BRAF* mutation had a similarly poor 5-year survival rate as MSS cancers with a *KRAS* mutation [112]. Additionally, a study that analysed survival of *KRAS* and *BRAF* mutant metastatic cancers following complete liver resection found that there were comparable overall survival rates although fewer data for *BRAF* mutant cancers was available [113].

Although the presence of CIMP has correlated with worse survival in MSS cancers compared to those without CIMP, the negative effect of the *BRAF* mutation far exceeded that of CIMP which suggested that the *BRAF* mutation was acting independently of CIMP [5]. Additionally, CIMP-high tumours with a *BRAF* mutation had a worse survival rate compared to CIMP-high with wild-type *BRAF*, which further implicates the *BRAF* mutation as a determining factor in detrimental patient outcome in microsatellite stable cancers [5].

## 8. Clinical Relevance of the *BRAF* Mutation in the Serrated Pathway

There is a continuum of tumourigenesis from a *BRAF* mutant sessile serrated adenoma that methylates *MLH1* at the point of dysplasia which then can rapidly progress to a *BRAF* mutant/MSI cancer [59, 114]. Alternatively, the clinical and molecular similarities occurring between advanced *BRAF* mutant traditional serrated adenomas (TSAs) and *BRAF* mutant/MSS cancers, such as similar gender distribution, propensity for CIMP, and mutant *p53* [9, 10, 54, 55], strengthen the proposal that TSAs most likely serve as precursor lesions for *BRAF* mutant/MSS cancers [54] (Figure 2).

Although the overall occurrence of *BRAF* mutant/MSS cancers is only 7%, a disproportionately large number of deaths would result due to their aggressive nature and poor survival. Stage II cancers typically do not receive adjuvant therapy. However, reports of stage II *BRAF* mutant/MSS cancers having shorter survival rates than *BRAF* wild-type/MSS cancers [4, 5, 79] suggests that the former have a higher risk of relapse. This may further highlight the relevance of screening for presence of a *BRAF* mutation and considering a more aggressive treatment strategy for stage II *BRAF* mutant/MSS cancers to reduce their associated survival risk.

With further understanding and classification of biomarkers of CRC, individualised therapy is being enhanced. *KRAS*, the upstream effector of *BRAF*, is recognized as a biomarker of resistance to anti-*EGFR* monoclonal antibodies (cetuximab and panitumumab) [115–117]. Furthermore, amongst patients treated with anti-*EGFR* therapy, decreased overall and progression-free survival has been associated with patients harbouring mutant compared to wild-type *KRAS* [118–120].

As *BRAF* is a downstream target in the MAPK pathway and has biologically similar activation as *KRAS*, studies have evaluated whether the *BRAF* mutation could also be considered a biomarker for anti-*EGFR* monoclonal antibody treatment. Several studies observed that similarly to *KRAS*, wild-type *BRAF* is also necessary for a response to this therapy as patients with mutant *BRAF* do not benefit from this treatment type [14, 15, 121, 122]. A meta-analysis of advanced *BRAF* mutant cancers treated with either cetuximab or panitumumab plus chemotherapy found that anti-*EGFR* therapy did not influence survival benefit compared to control regimens [123].

However, it has been found that a *BRAF* mutation does not confer a significant predictive role for anti-*EGFR* therapies when used in combination with chemotherapy compared to chemotherapy alone [124, 125]. Patients with a *BRAF* mutation had similarly poor survival rates either with or without the addition of anti-*EGFR* therapy, and therefore, it was concluded that the *BRAF* mutation had a negative prognostic rather than predictive role [118]. A study that found no significant survival benefit for patients with mutant *BRAF* treated with cetuximab concluded that the relatively low frequency of the *BRAF* mutation and its strong correlation with negative patient outcome may be contributing to the lack of significant difference in response to anti-*EGFR* treatment strategies and that larger patient cohorts are required to assess the predictive significance of mutant *BRAF* [125, 126].

Overall, it is recommended that the *BRAF* mutational status be confirmed at the time of both primary and metastatic cancer due to the distinctive biological subtype it confers, its poor prognostic association particularly in a MSS background, and that the majority of available data suggest that response to anti-*EGFR* therapy is less likely in mutant compared to wild-type *BRAF* cancers (Australian Cancer Guidelines 2017; http://www.cancer.org.au). Further testing and larger study populations may ultimately confirm *BRAF* mutational status as an additional and much needed biomarker for colorectal cancer and ensure its inclusion in routine screening outside of clinical trial settings.

## 9. Therapeutic Strategies for Mutant *BRAF*

Due to the aggressive nature of advanced *BRAF* mutant cancer, 60% of patients with *BRAF* mutant advanced cancer are less likely to be able to receive second-line chemotherapy [101, 127]. Therefore, intensive first-line FOLFOXIRI chemotherapy (consisting of a triple combination of 5-fluorouracil, irinotecan, and oxaliplatin) may be an efficient therapy approach as it showed improved response rates compared to administration of FOLFIRI (consisting of 5-fluorouracil and irinotecan only) in late-stage *BRAF* mutant cancer [127, 128]. Further, the inclusion of an anti-angiogenesis (anti-VEGF) monoclonal antibody, bevacizumab, to FOLFOXIRI treatment of BRAF mutant metastatic CRC did improve both progression-free and overall survival, but possibly due to the small sample sizes, statistical significance was not reached [129, 130].

There has been an emerging array of small molecule inhibitors that target mutant *BRAF* as well as key components of the upregulated MAPK pathway. Sorafenib was the initial molecular inhibitor of RAF-kinases developed; however, its clinical effects were limited due to its being a multitargeted kinase inhibitor that had greater affinity for other kinases besides BRAF [131].

The development of a small molecule inhibitor that selectively targets the *BRAF* V600E mutation, vemurafenib (PLX4032), resulted in regression of *BRAF* mutant advanced melanoma [132]. Although this response diminished after approximately 7 months [133], it confirmed *BRAF* V600E as a viable therapeutic target. However, similarly positive responses were not seen when advanced *BRAF* mutant colorectal cancer patients were treated with vemurafenib [134]. This reflects a partially different biological activity of the *BRAF* mutation depending on the cancer type and suggests that unique mechanisms of resistance may be activated in colorectal cancer compared to melanoma.

Dabrafenib is a potent and selective BRAF inhibitor that has shown over 100-fold selectivity for mutant compared to wild-type BRAF [131]. However, like vemurafenib, its clinical effect as a single agent in colorectal cancer is limited due to the rapid acquisition of resistance mechanisms. BRAF inhibition can induce direct feedback activation of EGFR that allows continued MAPK signalling and cellular proliferation [135]. Interestingly, melanoma cells do not express EGFR to the same extent as colorectal cancer cells, which explains in part the difference in clinical response seen from application of vemurafenib between the two cancer types [135]. In addition, upon BRAF inhibition, there is ERK-dependent activation of EGFR and subsequent reactivation of the MAPK pathway via CRAF, as well as dimerization between CRAF and BRAF ensuring pathway resignalling, and this renders BRAF inhibitors that act to sequester a BRAF mutant monomer, ineffective [24, 136].

Further mechanisms of MAPK pathway acquired resistance to single agent inhibition include ERK-mediated gain-of-function MEK1 mutations, BRAF amplification, and KRAS alterations [137, 138]. Crosstalk between pathways and upregulation of the PI3K signal can occur due to KRAS-mediated activation of PIK3CA [139].

The abundance of resistance mechanisms highlights the importance for combinatorial therapy approaches to attempt to mitigate pathway reactivation and disease relapse. Currently, there are several investigations underway testing the combination of inhibitors of MAPK pathway components such as BRAF and/or MEK, with anti-EGFR monoclonal antibodies and/or chemotherapeutic agents. A treatment approach of vemurafenib, cetuximab, and irinotecan for *BRAF* mutant advanced CRC had a 30% response rate and was recently included in the US National Comprehensive Cancer Network Guidelines for CRC [140]. A recent study investigating the combination of BRAFi (dabrafenib), MEKi (trametinib), and EGFRi (panitumumab) found a promising response rate of 21% and importantly a substantial reduction in phospho-ERK which demonstrated its efficacy [141]. Interestingly, serial monitoring of patients' plasma cell-free DNA (cfDNA) identified that rebounds in BRAF^V600E^ levels coincided with disease progression, as well as the emergence of adaptive resistance mechanisms such as mutant *KRAS*, which indicates cfDNA monitoring may correlate with patient response and influence combative therapy prescription, more so than CEA levels which is the typical clinical CRC marker.

ERK inhibitors have been found to resensitize *BRAF* mutant cancer cells that have acquired resistance to either BRAF or MEK inhibitors [142]. Recently, a phase I trial of the first in class ERK inhibitor, ulixertinib, resulted in anti-cancer effects in a variety of advanced *BRAF* mutant cancers, including CRC, which had previously failed MEK-ERK therapy [143]. Further studies including combinatorial approaches with this ERK inhibitor would be warranted.

Crosstalk activation of the PI3K pathway via signalling through KRAS as well as mutations in PIK3CA and PTEN that occur in 17% and 21% of BRAF mutant cancers, respectively [73, 144], can render cancer cells resistant to MAPK inhibition [145]. A phase II study investigating the combination of a PI3K*α* inhibitor (alpelisib) with a BRAF inhibitor (encorafenib) and EGFRi (cetuximab) showed a moderate response rate compared to dual therapy without alpelisib in heavily pretreated patients, which indicates that this approach may show clinical potential.

### 9.1. BRAF Mutant/MSI Cancers and Immunotherapy

As previously discussed, BRAF mutant/MSI have an activated immune profile due to the hypermutated phenotype and presence of multiple antigens that promote lymphocytic infiltration. The recent emergence of immune checkpoint therapy has had significant success in cancer types that display an active immune phenotype. A phase II study of the anti-PD1 antibody, pembrolizumab, showed substantial efficacy in MSI CRC with increased progression-free survival, whereas no response was found in MSS cancers [67]. A more recent investigation of nivolumab (anti-PD1) monotherapy and in combination with ipilimumab (anti-CTLA4) resulted in very promising response rates in patients with MSI CRC, especially with dual therapy, but again no response was noted in MSS cancers [146]. Further studies that address the efficacy of immune checkpoint therapy specifically in relation to presence of a BRAF mutation in a background of MSI would be warranted [147].

## 10. Conclusion

The presence of the *BRAF* V600E mutation in colorectal cancer influences clinical presentation, histology, molecular parameters, and patient outcome. Often, these factors are dependent on the genetic background of the cancer with the *BRAF* mutation occurring in microsatellite stable cancers conferring a significantly more aggressive phenotype. Investigations have found this latter type of colorectal cancer to be a distinct subgroup of the serrated pathway with unique molecular features. Additional aberrations such as epigenetic deregulation of key genes may be contributing to its advanced presentation. Further studies are required to assess the utility of the *BRAF* V600E mutation as a biomarker for colorectal cancer and to fully exploit its predictive, prognostic, and therapeutic potential.
